# Defensive Medicine in an Emergency Department: The Overuse of High-Sensitivity Cardiac Troponin I Testing

**DOI:** 10.3390/life14121563

**Published:** 2024-11-28

**Authors:** Mohammed Hani Sayyad, Nir Levi, Sharon Bruoha, Todd Zalut, Louay Taha, Mohammad Karmi, Nimrod Perel, Tomer Maller, Netanel Zacks, Maayan Sherm, Noam Fink, Pierre Sabouret, Khurram Nasir, Sarit Bar-Sheshet, Michael Glikson, Elad Asher

**Affiliations:** 1 Emergency Department, Shaare Zedek Medical Center, Faculty of Medicine, The Hebrew University of Jerusalem, Jerusalem 9112001, Israel; mohammeds@szmc.org.il (M.H.S.); er@szmc.org.il (T.Z.); 2 Jesselson Integrated Heart Center, Shaare Zedek Medical Center, Eisenberg R&D Authority and Faculty of Medicine, The Hebrew University of Jerusalem, 12 Shmuel Beit Street, Jerusalem 9103102, Israel; levin@szmc.org.il (N.L.); loayt@szmc.org.il (L.T.); mkarmi@szmc.org.il (M.K.); n_perel@yahoo.co.in (N.P.); tomer.maller@gmail.com (T.M.); netanelz@szmc.org.il (N.Z.); maayanshr@szmc.org.il (M.S.); mglikson@szmc.org.il (M.G.); 3 Department of Cardiology, Barzilai University Medical Center, Faculty of Health Sciences, Ben-Gurion University of the Negev, Ashkelon 7830604, Israel; sharonitob@gmail.com; 4 Assuta Medical Centers, Tel Aviv 6329302, Israel; 5 Department of Military Medicine, Faculty of Medicine, The Hebrew University of Jerusalem, Jerusalem 9190500, Israel; 6 ACTION Study Group, Institut de Cardiologie, Hôpital Pitié-Salpêtrière, Sorbonne Université, 75005 Paris, France; 7 National College of French Cardiologists, 13 Rue Niepce, 75014 Paris, France; 8 Houston Methodist DeBakey Heart and Vascular Institute, Houston Methodist Hospital, 6550 Fannin St., Houston, TX 77030, USA; 9 Clinical Laboratory Division, Shaare Zedek Medical Center, Jerusalem 9103102, Israel; saritbs@szmc.org.il

**Keywords:** troponin, emergency department, defensive medicine

## Abstract

Introduction: Cardiac troponin I is routinely measured in patients with suspected acute coronary syndrome. However, when a high-sensitivity cardiac troponin I (hs-cTnI) test is ordered without a clear clinical indication, unexpectedly elevated levels can lead to unnecessary diagnostic workups and inappropriate management. This study aimed to investigate physicians’ rationale for performing hs-cTnI tests in an emergency department (ED). Methods: In this prospective study, 1890 patients who underwent hs-cTnI measurement during their stay in an ED were included. Upon arrival, patients were classified into two groups based on their chief complaints: cardiac (36.6%) and non-cardiac (63.4%). Forty-seven ED physicians were asked to complete a questionnaire to assess their perspectives on the use of high-sensitivity cardiac troponin I (hs-cTnI) testing in the ED. Results: Out of the 47 ED physicians who responded to the questionnaire (94% response rate), 97.9% indicated that the purpose of hs-cTnI testing in the ED was to diagnose an acute cardiac event. However, 38.3% reported ordering hs-cTnI tests in non-cardiac patients due to medicolegal concerns. Additionally, 53% admitted to working under medicolegal pressure, and 50% believe they would have ordered fewer hs-cTnI tests if not for this medicolegal threat. Conclusions: defensive medicine is prevalent among ED physicians, and routine use of hs-cTnI testing as part of an evaluation can be explained in part by concern about liability and defensive medicine.

## 1. Introduction

A high-sensitivity cardiac troponin I (hs-cTnI) test is used both as a diagnostic and prognostic marker in acute coronary syndrome (ACS) patients [1,2], allowing for the detection of even minor myocardial infarctions [3,4,5]. However, the increased sensitivity of hs-cTnI comes at the cost of reduced specificity. Elevated troponin levels can also be observed in non-cardiac conditions, such as renal failure, hypertensive emergency, ischemic stroke, subarachnoid hemorrhage, and cor-pulmonale, and in systemic illness like sepsis, even in the absence of coronary artery disease. Furthermore, elevated troponin may occur in healthy individuals after strenuous exercise or, rarely, due to analytical interference from circulating antibodies with an hs-cTnI immunoassay (true false positive) [6,7,8]. As a result, elevated troponin levels are sometimes detected unexpectedly, underscoring the need for judicious interpretation across different populations and disease states [9,10,11,12,13,14]. Current guidelines from the European Society of Cardiology and the American College of Cardiology recommend hs-cTnI testing in the emergency department (ED) for patients presenting with suspected ACS [15,16,17]. However, the increasing availability and relatively low costs of hs-cTnI assays have led to their expanded use beyond guidelines’ recommendations. While this broader application can result in unnecessary diagnostic workups, hospitalizations, and increased resource utilization, potentially leading to a significant economic burden on healthcare systems [18], it is essential to recognize that testing outside of strict guideline conditions can also identify unexpected cardiac issues. Thus, careful considerations, informed by clinical suspicion and context, are needed when deciding to test for cardiac troponin in patients with non-cardiac symptoms [19,20].

Defensive medicine, which is a strategy employed by healthcare professionals globally, particularly physicians, involves the excessive and unnecessary utilization of diagnostic tests and interventions to mitigate potential risks and lawsuits [21,22]. However, this practice often detracts practitioners from adhering to evidence-based medicine principles, leading to both positive and negative outcomes, with potential side effects from futile interventions [23]. Fear of malpractice litigation contributes significantly to variability in ED decision-making, resulting in increased hospitalizations of low-risk patients and overuse of resources [24]. Many tests originally performed out of concern for liability may become so ingrained in customary practice that physicians may be unaware of their original motivation and could over time begin practicing unconscious defensive medicine [25].

The aim of this study was to investigate the rationale behind physicians’ decisions to perform hs-cTnI testing in the ED and to assess the extent of defensive medicine in the ED.

## 2. Methods

### 2.1. Study Design, Population, and Outcomes

A single-center, prospective study was conducted between 1 February 2023 and 28 February 2023 at the Shaare Zedek Medical Center, the largest ED in Jerusalem, Israel, serving an estimated population of 919,400 people. During the study period, 1890 patients who underwent at least one hs-cTnI test during their stay in the ED were included, of whom 1006 (53.2%) were males, with a mean age of 63.5 (±20.7) years.

Based on the chief complaint as recorded by the treating physician on admission, all study patients were categorized into two groups: cardiac complaints (chest pain, dyspnea, palpitations, or epigastric pain) and non-cardiac complaints. A total of 692 (36.6%) patients presented with cardiac complaints, while 1198 (63.4%) presented with non-cardiac complaints. Patients under 18 years old and pregnant women were excluded from the study.

For each group, the number of diagnostic tests, length of stay in the ED, admission rates, and in-hospital cardiac and non-cardiac mortality were determined. A positive hs-cTnI test was defined as hs-cTnI levels above the 99th percentile upper reference limit for the specific assay used (ARCHITECT STAT High-sensitivity Cardiac Troponin I assay, Abbott Diagnostics^®^, Lake Forest, IL, USA).

At the end of February 2023, after collecting all data regarding the hs-cTnI tests, each ED physician was required to complete a questionnaire (Table 1) to determine their reason for performing hs-cTnI testing and its impact on patient management. Physicians and nurses were blinded to the study’s purpose, except for the ED Director.

This study complied with the Declaration of Helsinki and was approved by the Institutional Review Board at the Shaare Zedek Medical Center (IRB protocol number 0003-23-SZMC). Patient data were de-identified prior to analysis. The trial received no external funding, and participants were not compensated for their involvement in the study. Informed consent was waived by the IRB due to the observational design of the study.

### 2.2. Data Collection

Baseline characteristics, including patient demographics, medical history, and medical treatment, were collected from patients’ electronic medical records (EMRs), in addition to data on the reason of their ED visit, time of discharge, or admission to inpatient departments. The number and value of hs-cTnI tests were collected and analyzed for each group. Data regarding in-hospital mortality and cause of death were retrieved from patients’ EMRs and the Israeli Population Registry. All data retrieved from questionnaires were gathered and analyzed.

### 2.3. Statistical Analysis

Results are presented as mean ± standard deviation (SD) for continuous variables with a normal distribution, as median and interquartile range (IQR) for continuous variables with a skewed distribution, and as counts and percentages for categorical data. A *t*-test was used to compare continuous variables, and for skewed distributions, a Mann–Whitney U test was applied. A chi-square test and Fisher’s exact test were used for categorical data. Binary logistic regression was employed to identify factors associated with the composite outcome. A two-tailed *p*-value of <0.05 was considered statistically significant. Statistical analysis was performed using SPSS software (version 21.0).

## 3. Results

### 3.1. Patients’ Characteristics

Patients with cardiac complaints were younger (60.7 ± 20.1 vs. 65.1 ± 20.9 years old; *p* < 0.001), had higher rates of prior ischemic heart disease (10.7% vs. 8%; *p* = 0.05), were more likely to be smokers (5.9% vs. 2.5%; *p* < 0.001), and had higher rates of dyslipidemia (19.8% vs.15.8%; *p* = 0.026). Additional baseline characteristics are presented in Table 2.

### 3.2. Troponin Levels

A total of 2427 hs-cTnI tests were performed during the study period in the ED. Of these, 1031 (42.5%) were performed in patients with cardiac chief complaints, and 1396 (57.5%) were conducted in patients with non-cardiac complaints. Patients with cardiac complaints had a higher rate of hs-cTnI levels > 99th percentile (21.2% vs. 17.2%; *p* = 0.03) and significantly higher hs-cTnI levels compared to patients with non-cardiac complaints (308.1 ± 2466.2 vs. 36.3 ± 287.3 ng/L, *p* < 0.001). Additionally, patients with cardiac complaints had higher hemoglobin levels (13.5 ± 2 vs. 12.9 ± 2.4 g/dL; *p* < 0.001) and lower lactate levels (1.6 ± 1.1 vs. 1.8 ± 1.7 IU; *p* = 0.016). Additional laboratory tests on arrival to the ED are presented in Table 3.

### 3.3. Hospitalization Course and Mortality Rates

The mean length of stay in the ED was shorter for patients with cardiac complaints compared to those with non-cardiac complaints (8.6 ± 8.3 vs. 10.8 ± 12.3 h; *p* = 0.001). Patients with cardiac complaints were more likely to be admitted to the intensive cardiovascular care unit (ICCU) (5% vs. 1.2%, *p* < 0.001) or cardiology ward (11.4% vs. 2.6%, *p* < 0.001). In contrast, patients with non-cardiac complaints were more likely to be admitted to the internal medicine department (18.7% vs. 8.3%, *p* < 0.001) or the general intensive care unit (0.5% vs. 0.1%, *p* < 0.001).

Percutaneous coronary intervention (PCI) was performed in 45 (52%) of the 87 patients who underwent coronary angiography in the cardiac symptoms group, compared to 3 (20%) of the 15 patients in the non-cardiac symptoms group.

Patients with cardiac complaints had lower in-hospital and 30-day all-cause mortality rates (1.6% vs. 3.1%; *p* = 0.037 and 2.3% vs. 4%; *p* = 0.05, respectively). Additional in-hospital outcomes are presented in Table 4.

### 3.4. ED Physicians’ Characteristics and Perspectives on hs-cTnI Measurement

Forty-seven (94%) ED physicians who worked during the study period, including internal medicine residents working evening, night, and weekend shifts, responded to the questionnaire. Of them, 61.7% were male, and 44.7% were aged 30–39 years. The majority (68%) were residents, and 19.1% were board-certified in Emergency Medicine (Table 1).

Most physicians stated that the primary reason for ordering hs-cTnI tests in the ED was to diagnose acute MI. However, 38.3% reported that hs-cTnI tests were ordered in non-cardiac patients due to medicolegal concerns. Nearly one-third of the physicians believed that over 10,000 hs-cTnI tests were ordered in the ED each month, though the actual number was 2427 (Table 1). Additionally, 53% acknowledged practicing under medicolegal pressure, whereas 55% indicated they would have ordered fewer hs-cTnI tests if they had not been working under the threat of medical malpractice (Table 1).

## 4. Discussion

The main findings of our study are as follows: (1) Defensive medicine is highly prevalent among ED physicians, with almost 40% admitting to practicing it; (2) more than 35% of hs-cTnI tests were performed in patients without cardiac symptoms; (3) half of physicians stated they would have ordered fewer hs-cTnI tests if not for medicolegal threats; and (4) nearly one-third of physicians believed the number of hs-cTnI tests ordered each month exceeded 10,000.

Defensive medicine was notably common in our study, with almost half of the ED physicians reporting that they ordered hs-cTnI tests without clinical indication. Additionally, nearly 20% of the physicians requested hs-cTnI tests without a documented cardiac indication (Table 1). Interestingly, the reason for ordering unnecessary blood tests may not always be driven by a conscious concern about liability, especially when the test is commonly prescribed. In a study evaluating the rationale for routine erythrocyte sedimentation rate (ESR) testing among internal medicine residents, the most common reason cited (47.7%) was “to identify severe or hidden diseases” [25]. The authors hypothesized that this unnecessary test ordering was partially driven by “unconscious” defensive behavior. Procedures initially performed out of fear of litigation may become so ingrained in routine practice that physicians no longer recognize the original motivation for them. This aligns with our findings as many ED physicians ordered hs-cTnI tests without a valid clinical reason or suspected diagnosis.

We also observed that more than 35% of hs-cTnI tests were performed on patients without cardiac symptoms. The ease of ordering blood tests in hospital settings and the potential consequences of missing a life-threatening diagnosis often lead to automatic test ordering regardless of the clinical scenario. Defensive practices can also be driven by conscious concerns over malpractice liability [25].

The high rate of unnecessary tests in our study is in accordance with previous studies [22,26], indicating that defensive medicine is a widespread practice [27]. For instance, a Japanese survey among gastroenterologists found that 98% of respondents admitted to practicing defensive medicine [28]. This behavior is particularly common in medical specialties with higher risks and in healthcare systems where legal actions are more frequent [29,30].

Furthermore, defensive behavior is more prevalent among physicians in private practice compared to those in government positions [31]. Notably, defensive medicine is influenced more by a physician’s confidence in their liability coverage than by their professional experience. Physicians lacking confidence in their liability coverage were more than twice as likely as their peers to order unnecessary diagnostic tests [29]. This finding supports our results, where nearly 50% of ED physicians stated they would have ordered fewer hs-cTnI tests if not for medicolegal threats, underscoring the significant impact of defensive medicine on modern clinical practice. Interestingly, the fact that nearly a third of the ED physicians believed that over 10,000 hs-cTnI tests were performed monthly, while the actual number was 2427, highlights the level of concern about medicolegal liability (Table 1).

Although the financial costs of defensive behavior were not addressed in this study, they represent a major negative consequence of this practice. Defensive medicine can lead to overtreatment, overdiagnosis, prolong hospital stays, and ultimately damage trust between healthcare providers and patients [32,33].

Finally, the differences in length of hospital stay and mortality rates between cardiac and non-cardiac complaint patients were notable and warrant further discussion. We believe that part of the explanation lies in the baseline characteristics of the cohorts. For example, patients presenting with non-cardiac symptoms were generally older and had more comorbidities, such as higher rates of dyslipidemia, a history of ischemic heart disease, and atrial fibrillation (Table 1). These factors likely contributed to their longer hospital stays and higher mortality rates. Furthermore, patients with cardiac complaints were typically admitted to specialized units, such as a cardiology ward or coronary care unit, whereas those with non-cardiac complaints were more often admitted to internal medicine or general intensive care units. It is plausible that focused, specialized care in cardiac units may lead to better outcomes compared to more generalized care, which could account for the observed differences.

## 5. Limitations

The study had several limitations. First, it was conducted in the ED of a single public university medical center, which may limit the generalizability of our findings to other healthcare settings. Additionally, the study was conducted over a one-month period, which may not capture broader trends. Finally, most respondents were physicians at various stages of their training, which may not reflect the full extent of defensive medicine across different healthcare contexts.

## 6. Conclusions

Defensive medicine is highly prevalent among ED physicians, potentially leading to misdiagnoses and an increase in invasive procedures, which can be associated with serious adverse events. Enhanced education on the appropriate use of hs-cTnI, adherence to clinical guidelines, and careful interpretation of test results in the context of each patient’s condition are essential for healthcare providers in the ED to ensure that diagnostic tests like hs-cTnI are ordered appropriately.

## Figures and Tables

**Table 1:** High-sensitivity cardiac troponin I survey for emergency department physicians.

Question	Answer Options
Gender	Male: 61.7%, Female: 38.3%
Age	18–29: 36.2%, 30–39: 12.8%, 40–49: 44.7%, 50+: 6.4%
Training Level	Intern: 12.8%, Resident: 68.1%, Board-certified ED Physician: 19.1%, Manager: 0%
Main reason for ordering hs-cTnI tests in the ED	Routine test: 0%, Diagnose acute cardiac issue: 97.7%, Fear of malpractice: 0%, Don’t know: 2.3%
Reason for ordering hs-cTnI tests for patients without cardiac symptoms	Routine test: 17%, Diagnose acute cardiac issue: 27.7%, Fear of malpractice: 38.3%, Don’t know: 17%
Do you document the rationale for ordering hs-cTnI tests in the medical record?	Only when suspecting MI: 34%, Always document: 34%, Usually don’t document: 32%
Do you feel that part of your work in the ED is influenced by fear of malpractice lawsuits, rather than purely clinical considerations?	Hardly ever: 17%, Slightly: 29.8%, Significantly: 36.2%, Very much: 17%
Do you think hospital policies are driven by a desire to avoid malpractice lawsuits, regardless of medical necessity?	Hardly ever: 31.9%, Slightly: 38.3%, Significantly: 23.4%, Very much: 6.4%
If there was no concern about malpractice lawsuits, do you think fewer hs-cTnI tests would be ordered?	No: 36.2%, Yes, 20% less: 21.3%, Yes, 30% less: 27.7%, Yes, 50% less: 8.8%
In your opinion, how many hs-cTnI tests are performed in the ED monthly?	1–100: 5%, 100–1000: 10.6%, 1000–10,000: 61.7%, 10,000–50,000: 27.7%, 50,000–500,000: 5%

hs-cTnI, high-sensitivity cardiac troponin; ED, emergency department.

**Table 2:** Baseline patient characteristics.

Parameter	Overall n = 1890	Cardiac n = 692	Non-Cardiac n = 1198	*p* Value
Age (years), mean ± SD	63.5 ± 20.7	60.7 ± 20.1	65.1 ± 20.9	<0.001
Male Gender, %	1006 (53.2)	388 (56.1)	618 (51.6)	0.06
Past/Active smoker, %	71 (3.7)	41 (5.9)	30 (2.5)	<0.001
Hypertension, %	501 (26.5)	196 (28.3)	305 (25.5)	0.174
Diabetes Mellitus, %	125 (6.6)	52 (7.5)	73 (6.1)	0.231
Dyslipidemia, %	326 (17.2)	137 (19.8)	189 (15.8)	0.026
Ischemic Heart Disease, %	170 (9)	74 (10.7)	96 (8)	0.05
Atrial Fibrillation, %	190 (10)	54 (7.8)	136 (11.3)	0.013
Prior Stroke/TIA, %	34 (1.8)	11 (1.6)	23 (1.9)	0.603

SD, standard deviation; TIA, transient ischemic attack.

**Table 3:** Laboratory tests on admission.

Parameter	Overall n = 1890	Cardiac n = 692	Non-Cardiac n = 1198	*p* Value
Lactate (IU), mean ± SD	1.7 ± 1.6	1.6 ± 1.1	1.8 ± 1.7	0.016
Hemoglobin (g/dL), mean ± SD	13.2 ± 2.3	13.5 ± 2	12.9 ± 2.4	<0.001
CRP (mg/dL), mean ± SD	3.2 ± 5.9	2.1 ± 4.6	3.7 ± 6.4	<0.001
hs-cTnI (ng/L), mean ± SD	135.8 ± 1514.7	308.1 ± 2466.2	36.3 ± 287.3	<0.001
Time (hours) from admission to first hs-cTnI, mean ± SD	1.5 ± 2.8	1.3 ± 1.3	1.6 ± 3.3	0.061
* Positive hs-cTnI (%)	353 (18.7)	147 (21.2)	206 (17.2)	0.03
Time from admission to second hs-cTnI, mean ± SD	13.9 ± 29.2	9.7 ± 26	19.3 ± 32.2	<0.001
Delta hs-cTnI (ng/L) (first to second), mean ± SD	267.3 ± 2846.1	448.7 ± 3777.7	33.4± 263	0.064

SD, standard deviation; IU, international units; CRP, C-reactive protein; hs-cTnI, high-sensitivity cardiac troponin I. * Positive test was defined as hs-cTnI above the 99th percentile upper reference limit in at least one of two measurements.

**Table 4:** Patients’ outcomes.

Parameter	Overall n = 1890	Cardiac n = 692	Non-Cardiac n = 1198	*p* Value
Length of Hospital Stay (days), mean ± SD	9.5 ± 14.5	6.3 ± 6.7	10.9 ± 16.6	<0.001
Home Discharge from ED, %	1302 (68.9)	511 (73.8)	791 (66)	
Length of Stay in ED (h), mean ± SD	10 ± 11.1	8.6 ± 8.3	10.8 ± 12.3	0.001
Cardiac Catheterization, %	102 (5.4)	87 (12.6)	15 (1.2)	<0.001
ED mortality, %	6 (0.32)	1 (0.14)	5 (0.42)	0.31
In-Hospital Mortality, %	49 (2.6)	11 (1.6)	38 (3.2)	0.037
30-Day Mortality, %	64 (3.4)	16 (2.3)	48 (4)	0.05

SD, standard deviation; ED, emergency department; ICCU, intensive coronary care unit; ICU, intensive care unit.

## Data Availability

The original contributions presented in the study are included in the article, further inquiries can be directed to the corresponding author.
